# *In Silico* Modelling of Tumour Margin Diffusion and Infiltration: Review of Current Status

**DOI:** 10.1155/2012/672895

**Published:** 2012-07-11

**Authors:** Fatemeh Leyla Moghaddasi, Eva Bezak, Loredana Marcu

**Affiliations:** ^1^Department of Medical Physics, Royal Adelaide Hospital, North Terrace, Adelaide, SA 5000, Australia; ^2^School of Chemistry and Physics, The University of Adelaide, North Terrace, Adelaide, SA 5000, Australia; ^3^Faculty of Sciences, University of Oradea, Oradea, Romania

## Abstract

As a result of advanced treatment techniques, requiring precise target definitions, a need for more accurate delineation of the Clinical Target Volume (CTV) has arisen. Mathematical modelling is found to be a powerful tool to provide fairly accurate predictions for the Microscopic Extension (ME) of a tumour to be incorporated in a CTV. In general terms, biomathematical models based on a sequence of observations or development of a hypothesis assume some links between biological mechanisms involved in cancer development and progression to provide quantitative or qualitative measures of tumour behaviour as well as tumour response to treatment. Generally, two approaches are taken: deterministic and stochastic modelling. In this paper, recent mathematical models, including deterministic and stochastic methods, are reviewed and critically compared. It is concluded that stochastic models are more promising to provide a realistic description of cancer tumour behaviour due to being intrinsically probabilistic as well as discrete, which enables incorporation of patient-specific biomedical data such as tumour heterogeneity and anatomical boundaries.

## 1. Introduction

**Figure 1:**
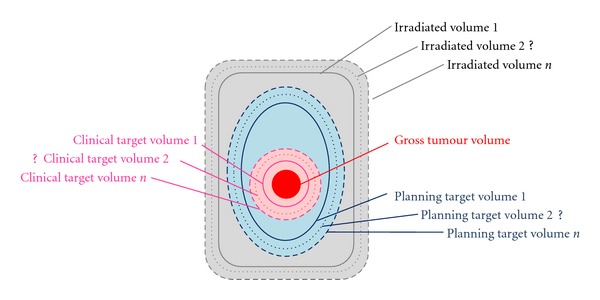
Schematic diagram of radiotherapy irradiation volumes.

Advanced radiotherapy techniques like 3D Conformal Radiotherapy (3D-CRT), Intensity-Modulated Radiation Therapy (IMRT), and Image-guided Radiation Therapy (IGRT) restrict the high dose region to defined target volumes to spare adjacent normal tissue. The margins are generally reduced for modern radiotherapy techniques due to (a) more accurate organ specification with the use of daily image guidance that results in minimization of set up error, and (b) superior conformity of dose distribution to irradiation target volumes. However, a successful implementation of these techniques, that is, achieving an acceptable Tumour Control Probability (TCP) and Normal Tissue Complication Probability (NTCP), requires very accurate target volume delineation. According to ICRU report 50, the “Clinical Target Volume (CTV) is a volume encompassing visible Gross Tumour Volume (GTV) and subclinical malignant disease” [1]. Since subclinical disease cannot be detected by imaging technologies, in contrast to gross tumour volume, which is the visible extent and location of malignant disease [1], CTV needs to be estimated. To ensure that CTV receives the prescribed dose, the Planning Target Volume (PTV) is drawn to account for several possible uncertainties. These uncertainties are due to both physiologic movements which are not controllable (e.g. patient's respiration) and to daily set-up variations. PTV is then the volume for which dose calculation is performed and ensures that the whole of CTV will receive the full prescribed radiation dose.  Figure 1 schematically illustrates radiotherapy irradiation volumes and their respective uncertainties regarding volume delineation.

**Figure 2:**
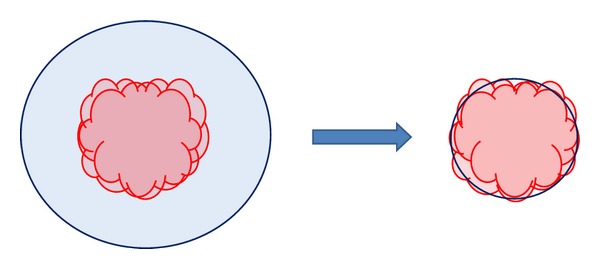
Schematic diagram of CTV and PTV correlation for conventional treatment techniques, on the left, as compared to modern treatment techniques, on the right. CTV is indicated by red contour and blue contour defines the PTV. As shown, the reduction of PTV may result in missing a part of microscopic disease that leads to poor treatment efficacy.

Among radiotherapy target volumes, delineation of the Clinical Target Volume (CTV) is the most controversial. To date, there is no consensus regarding the extent of histological disease, thus the question of how far CTV is extended beyond GTV is mostly left to the discretion of radiation oncologists based on their experience, depending on patient's histopathological data. The uncertainty in CTV represents a limitation on reduction of the irradiated target volume. When the irradiated target volume is reduced due to dose conformity of new treatment modalities, NTCP is improved. On the other hand, the issue of CTV fuzziness becomes a cause of concern because any PTV reduction enhances the risk of missing a part or a few cells of subclinical disease, as illustrated in Figure 2. It is worth mentioning that missing one single cell reduces TCP to 37%. (The Poisson distribution definition for TCP: TCP = *e* ^−*n*(*D*)^, where *n*(*D*) is the expected number of surviving clonogens.) Therefore, in order to confidently reduce the irradiated target volume, as is the trend with current treatment techniques, the pattern of microscopic extension needs to be known or predicted.

### 1.1. Biological Background

Normal growth and regeneration of an organ requires cells to undergo cell division and to proliferate. The rate of proliferation, however, is systematically regulated to ensure the balance between cell proliferation and cell loss as well as integrity and functionality of each organ. This regulation occurs at cell cycle check points where progression to a subsequent phase is prevented unless prerequisites are satisfied. DNA lesions are recognized at check points that lead onto repair pathways [4]. Normally, cells with unrepaired DNA cannot continue their cycle and are led to apoptosis (programmed cell death). Any uncontrolled proliferation of cells, ensuing a series of DNA mutations, results in abnormal aggregation of cells called a tumour. An evolving tumour population undergoes two stages, namely, *avascular phase* and *vascular phase* and transition between these two phases requires angiogenesis, a process which involves development and recruitment of blood vessels to supply tumour cells with nutrients [5, 6]. Tumour commences its growth primarily via cell proliferation in an avascular phase. Further in its growth, individual tumour cells secrete a substance called Tumour Angiogenesis Factor (TAF) that initiates angiogenesis [6]. At this stage, that is, the beginning of a vascular phase, tumour acquires the capability to invade locally in the adjacent normal tissue, and later tumour cells can detach themselves from the primary mass and migrate through blood or lymphatic system to other sites in the body to produce new colonies (i.e., metastasis) [6–9]. 

The Extracellular Matrix (ECM) is the external part of tissue on which cells reside. It provides structural support to the cells, regulates intercellular communications and so forth. The ECM also imposes spatial constraint on tumour proliferation. On the other hand, the tumour invasion is known to be facilitated by gradients in the ECM density (i.e., an ECM gradient is a directional rise in ECM density, and its magnitude determines how fast the ECM density rises in that direction). These gradients cause the cells in the outer layer of a tumour to break away from the primary tumour mass and move along the gradient, a phenomenon called *haptotaxis* [9]. It is known that, Matrix Degrading Enzymes (MDEs) produced by cancer cells degrade the surrounding ECM resulting in development of ECM gradients [9]. 

Apart from proliferation and haptotaxis, other factors like cell-cell adhesion, cell-matrix adhesion, and ECM density also affect cell motility in the course of tumour invasion [6].

In summary, tumour evolution is an interrelated multistage process that starts from a series of cancer-associated gene mutations leading to formation of a colony that could further invade adjacent tissues and finally metastasize in distant organs. Better understanding of biological mechanisms of cancer development helps to anticipate the behaviour of the tumour that undoubtedly leads to a better treatment efficacy.

### 1.2. Mathematical Modelling

Mathematical modelling is a suitable tool to generate algorithms to correlate information acquired from imaging techniques to the pattern of growth and tumour invasion. In a typical course of model development, biological phenomena are represented in mathematical equations. The solutions of the equations, in return, provide predictions of tumour evolution, tumour aggressiveness in a given patient, and so forth. The validity of a model is then examined by comparison with available actual data, and iteration is performed until an adequate match is reached and thus a plausible model is obtained. A semirealistic model developed in this manner provides an insight into biological mechanisms of tumour growth and invasion under a variety of circumstances. It also allows for assessment of potential treatment regimens. The model could be useful for clinicians in clinical tumour volume definition.

Oncogenesis can be modelled at three levels: (1) sub-cellular level, (2) cellular and microscopic level that concerns individual cell behaviour while taking into account cell-extracellular matrix (ECM) interactions, and (3) macroscopic level that is related to the evolution of tumour in terms of cell density and mostly is based upon reaction-diffusion equations [10].

In this paper, some of the recent computational and mathematical models developed for tumour growth and invasion are reviewed. Two approaches used for modelling, analytical and stochastic, are discussed individually in the following sections.

## 2. Deterministic Models

### 2.1. Analytical Models

Analytical modelling of tumour growth has been typically done based on the reaction-diffusion equations in the literature. Swanson et al. [11] reviewed some recent models developed for glioma of the brain. The problem was initially formulated as a conservation equation by Murray's group [12–14] as: the rate at which tumour cell population changes is equal to diffusion (motility) of tumour cells plus proliferation of tumour cells. For untreated glioma, this can be represented in a mathematical form as [11, 15]

(1) $$\begin{array}{c} \frac{\partial c}{\partial t}=-\nabla \cdot J+\rho c, \end{array}$$

where *c*(*x*, *t*) denotes the density of tumour at location *x* and time *t*, ∇·*J* is the diffusion component (i.e., outflow of material out of the system), and *ρc* is proliferation component (inflow of material in the system), where *ρ* is the proliferation coefficient. Using the Fick's first law that assumes the diffusive flux flows from high-concentration regions to low-concentration regions, the diffusion component is related to tumour cell density as follows:

(2) $$\begin{array}{c} J=-D\frac{\partial c}{\partial x}\overset{\text{in}\;3\text{D}}{\to }J=-D\nabla c. \end{array}$$

Thus (1) takes the form

(3) $$\begin{array}{c} \frac{\partial c}{\partial t}=\nabla \cdot \left(D\nabla c\right)+\rho c, \end{array}$$

where *D* is diffusion coefficient representing active motility of cancer cells and ∇ denotes spatial gradient operator. The first term, the diffusion component, is related to the periphery of the tumour while the second term, the proliferation component, pertains to active part of tumour core and is described by cellular proliferation laws (e.g., exponential growth) [10]. The assumptions considered in this model were the following.

- Brain tissue is homogeneous thus diffusion coefficient, *D*, is constant throughout the brain.
- Tumour growth is generally exponential thus *ρc* is constant.
- Boundary condition: *c*(*x*, 0) = *f*(*x*), where*f*(*x*) is initial profile of the tumour and there is no migration beyond brain boundaries.

Thus (3) reduces to

(4) $$\begin{array}{c} \frac{\partial c}{\partial t}=D\nabla^{2}c+\rho c. \end{array}$$

One of the consequences of (4) is that tumour density distribution, *c*, is a function of the ratio of *ρ*/*D* thus two different tumours whose different combinations of *ρ* and *D* result in the same ratio of *ρ*/*D*, appear the same at a single observation time. Hence, just a single MRI/CT image is not sufficient to estimate CTV correctly without knowing the pattern of tumour cell density distribution. 

A more realistic approach was taken by Swanson et al. [2, 17]who introduced the geometry of the brain into the model, thus in the revised form of the model, the following assumptions were considered.

(i) Complex geometry of brain is introduced, thus diffusion coefficient, *D*, is not uniform and is a function of location in the brain tissue.
(ii) Equation (3) is applied to describe the pattern of growth in diffusive models with *D* being a function of *x* as follows:
  (5) D(x)={DW,(diffusion  coefficientin   white  matter  of  the  brain),DG,(diffusion  coefficient  in   gray  matter  of  the  brain),         where  DW>DG.

**Figure 3:**
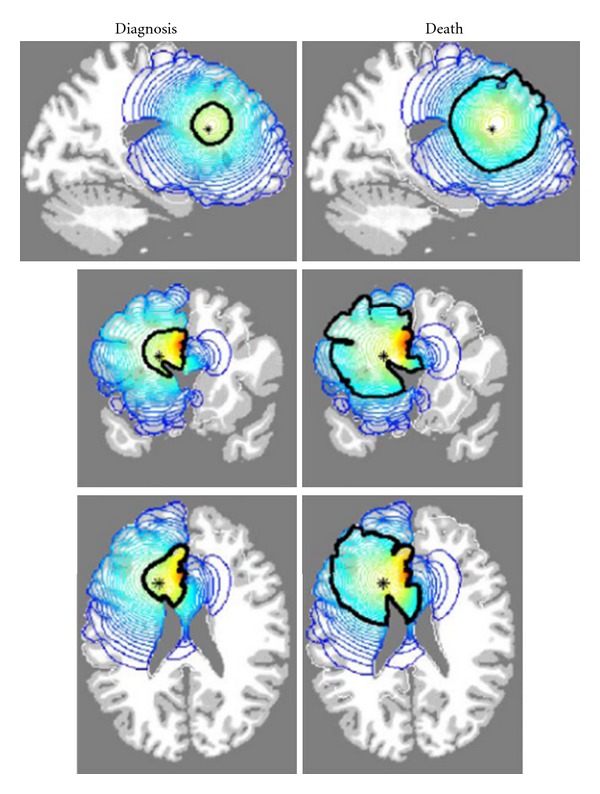
The left column corresponds to the tumour at diagnosis and right column corresponds to tumour at death. The dark black contour defines the detectable edge of tumour by (MRI), red contour indicates high density of tumour cells, and blue contour denotes low-density disease. Courtesy of Swanson et al. [2].

To determine the model parameters, 12 serial CT scans of a patient, diagnosed with astrocytoma, during his terminal year were examined to derive estimations for velocities of tumour margin advance through grey and white matter, *ν*_*G*_ and *ν*_*W*_, respectively. Fisher's approximation (*D* = *ν* ^2^/4*ρ*) was then applied to correlate velocity, *ν*, of detectable tumour margin with proliferation rate and diffusion coefficients. According to CT scans of the right hemisphere (predominantly grey matter), *ν*_*G*_ was identified to be 0.008 cm/day, thus Fisher approximation gives *D*_*G*_ = 0.0013 cm^2^/day, and *D*_*W*_ being almost five times of *D*_*G*_ becomes 0.0065 cm^2^/day. To assign diffusion coefficients to corresponding brain cells, spatial distribution of white and grey matter was adopted from the brain web database [31]. Applying these determined parameters in the simulation based on (3) describing virtual gliomas growth, two-dimensional plots of tumour cell density on coronal, sagittal and axial planes were generated, as shown in Figure 3. Using these plots, they determined the part of tumour volume that can be visualized using MRI technique. Enhanced MRI technique has a detection threshold of 400 cells/mm^2^. This means that any part of tumour having a concentration below this threshold is not detectable on a MRI image. The comparison between detectable part and simulated profile provides an insight into how far and at what concentration microscopic disease is invaded beyond visible tumour. This model that derived the behaviour of glioma according to two factors (“*D*” and “*ρ*”) demonstrates that the distribution of ME in invasive gliomas does not follow an isotropic pattern that is invariably assumed by clinicians for definition of CTV.

The biomathematical modelling based on (3) in conjunction with serial pre-treatment MRI images of the patient also provides a tool to quantify patient-specific proliferation and diffusion rates. Wang et al. [32] examined two pretreatment MRI images of each of a population of 32 patients diagnosed with Glioblastoma (GBM) to quantify patient-specific kinetic rates of glioma cells (net proliferation and diffusion rates). These parameters are used to predict the course of disease and, more importantly, to assess the efficacy of different treatment plans for each individual patient through a survival analysis. In the survival analysis, the effectiveness of any treatment was measured via the ratio of actual survival time after respective therapy to the calculated survival time (by the model) without therapy.

The evolution of mathematical modelling to gain insight into the mechanism of GBM growth and invasion initiated by Swanson et al. [11, 17] was followed by Stein et al. [20] who developed a continuum model and compared the outcome of the model with 3D *in vitro* experiments on the three dimensional pattern of growth of GBM spheroids. It was concluded that GBM spheroids consist of two classes of cells, namely, proliferating core cells and peripheral migrating cells. This finding was later included in other models like the model of Thalhauser et al. [22] in which three dependent variables, namely, the concentration of migrating cells, proliferating cells and oxygen (mmHg) were correlated in three partial differential equations for tumour development around a central blood microvessel. Analysis of the density distribution profiles of these two classes of cells led to a hypothesis regarding emergence of metastatic phenotype to occur for population of cells containing highly motile cells. This hypothesis is based on the evidence that populations of motile cells grow to lower densities compared to aggressive growers (mobile cells), and hence they are unlikely to cause vascular network collapse since they cause less compressive pressure on microvessel walls. In a more recent progress, Eikenberry et al. [8] incorporated haptotaxis in GBM models and also extended the model stochastically to form a deterministic-stochastic system for modelling. The mathematical model was developed based on four dependent variables: the concentration of migrating cells, proliferating cells, ECM, and matrix degrading enzyme. The system of partial differential equations was discretized to allow for stochastic estimation of the transition probability between proliferating and migrating class of cells at each grid point. The stochastic nature of the model allows for applying patient-specific geometry of brain and location of tumour inside the brain during simulation. The simulation was performed for an actual clinical case of a GBM patient undergoing a course of treatment including surgical resection, gamma knife, and chemotherapy. The model qualitatively reproduced the actual tumour growth of the patient. However, the model failed to simulate the deformation of surgical cavity. 

The spatial-temporal evolution of the brain tumour in the presence of chemotherapy was investigated by Tracqui et al. [2, 12]. Twelve successive CT scans during the terminal year of a patient diagnosed with astrocytoma were studied. The patient received two courses of chemotherapy during 12 months before death, thus (3) can be modified as

(6) $$\begin{array}{c} \frac{\partial c}{\partial t}=\nabla \cdot \left(D\nabla c\right)+f\left(c\right)-g\left(c\right), \end{array}$$

where *g*(*c*) is the cell loss due to chemotherapy and defined as

(7) $$\begin{array}{c} g\left(c\right)=\left[K_{1}\left(t\right)+K_{2}\left(t\right)\right]c \end{array}$$

with

(8) $$\begin{array}{c} K_{1}\left(t\right)=\{\begin{array}{cc} k_{1}, & \text{during the time the first course}\\ & \text{of drug was delivered,}\\ 0, & \text{during the time the second course}\\ & \text{of drug was delivered,} \end{array}\\ K_{2}\left(t\right)=\{\begin{array}{cc} 0, & \text{during the time the first course }\\ & \text{of drug was delivered,}\\ k_{2}, & \text{during the time the second course}\\ & \text{of drug was delivered,} \end{array} \end{array}$$

where *k*_1_ and *k*_2_ are positive constants. 

The proliferation term,*f*(*c*), is typically taken as a linear function of *c* (exponential proliferation) or a nonlinear function of *c* (logistic proliferation) when the proliferation is limited, since cell density is close to its maximum:

(9) $$\begin{array}{c} f\left(c\right)=\{\begin{array}{cc} \rho c, & \text{exponential proliferation,}\\ \rho c\left(1-c\right), & \text{logistic proliferation.} \end{array} \end{array}$$

The area of tumour was evaluated at each successive CT scan and then the data was compared to the values derived from (6). The comparison between time evolution of simulated tumour area and tumour areas acquired from CT scans showed a distinctive discrepancy, particularly before the end of the first course of chemotherapy. Consequently, the assumptions were revised and it was postulated that there is a second cell density *c*_2_(*x*, *y*, *t*) present which is resistant to the first course of chemotherapy but sensitive to the second course. The insensitivity of the second population was considered to be due to mutations from the radiotherapy administered three years earlier. Given this condition, the system was described mathematically as follows:

(10) ∂c1∂t=∇·(D∇c1)+ρ1c1(1−c)−[K1(t)+K2(t)]c1∂c∂t=∇·(D∇c)+ρ1c1(1−c)+ρ2c2(1−c)−K1(t)c1 −K2(t)c,

where *ρ*_1_ and *ρ*_2_ are proliferation rates corresponding to the first and second cell density, respectively, and variable *c* represents the total density of tumour cells (*c* = *c*_1_ + *c*_2_). 

After optimization and identification of unknown parameters, the identified values were found to be in agreement with known biological data (e.g., *D* = 1.2 × 10^−7^ cm^2^/s which is comparable with estimation of glioma cell migration rate obtained from *in vitro* experiments [33]).

Woodward et al. [15, 34] modified Tracqui's model for the same case study in terms of initial conditions related to distribution of type one and two of cancerous cells. In contrast to Tracqui's model that assumed an approximate initial distribution of 90% of type one and 10% of type two cancerous cells, Woodward included another parameter as the number of type one cells remaining after surgery followed by X-ray therapy 1000 days before the first scan and also assumed that type two cancerous cells are the result of mutations of type one cells three years earlier. This allowed for prediction of distribution of each type of cells at the time of diagnosis (rather than making a rough estimation) and at any time during the terminal year. Furthermore, the simulated evolution of the tumour was used to retrospectively evaluate different courses of treatments (e.g., different extent of surgical resections instead of chemotherapy) in terms of their respective subclinical recurrence.

Swanson et al. [11, 35] investigated the incorporation of cell loss due to chemotherapy in a more general formulation by defining *g*(*c*) to be a periodic function such that for the time periods chemotherapy is on, *g*(*c*) is equal to a specific positive constant, *k* (indicating the rate of cell loss due to chemotherapy), and otherwise is zero. The model was originally formulated assuming homogeneous drug delivery and further developed to take into account heterogeneity in drug delivery, whereby drug delivery is expected to be less in white matter compared to that in gray matter. The experimental observation of shrinkage of gliomas in specific areas together with persistent growth in other areas of the brain following chemotherapy was explained by this model. 

Clatz et al. [10] developed a numerical model to simulate the three-dimensional pattern of growth and invasion of Glioblastomas. To account for different diffusion coefficients which are dependent on the brain tissue, the anatomical atlas of the brain in conjunction with Diffusion Tensor Image (DTI) were employed. The algorithm comprised of four steps. First, the patient MRI images were registered on the brain atlas on which gross volumes were delineated by a radiation oncologist. In the second step, the image registered on atlas was used to produce patient's tetrahedral mesh of brain in which diffusion coefficients respective to each voxel were specified using brain atlas and DTI of the patient. Simulation was performed in the third step by applying reaction-diffusion equation on initial tetrahedral mesh of brain. Ultimately, to measure the validity of the model, the simulated profile was compared with brain deformation seen on the patient MRI images in six months later. 

Bondiau et al. [36] applied the virtual model of glioma growth developed by Clatz on actual data of a single patient and compared tumour growth pattern derived from the model with current radiotherapy margins. Tumour growth was studied in two scenarios, namely, high diffusion-low proliferation (HD-LP) and high proliferation-low diffusion (HP-LD) tumours. It was observed that, with 2 cm margin, 2.1% and 15.1% of microscopic invasive tumour cells fall outside margin in HP-LD and HD- LP tumours, respectively. Also 53.5% and 55.5% of cells inside margin in HP-LD and HD- LP, respectively, are normal brain cells. Therefore, it was concluded that uniform clinical margins may not be adequate to cover whole tumour neither to spare normal tissue. Although this conclusion is supported by many other studies, the rationale of this comparison is argued on the basis that a model which is based on a single patient clinical data, though sophisticated, cannot be considered as a criterion to assess clinical margins. It first needs to be validated against some actual clinical data (e.g., recurrence rate) in a statistically sufficient number of patients.

The effect of external beam radiation therapy was incorporated in the reaction-diffusion model in the study of Rockne et al. [23]. Therefore, the conservation of cells (3) can be modified as:

(11) ∂c∂t=∇·(D∇c)︸Diffusion  of  glioma  cells+ρc(1−ck)︸Logistic  proliferation+R(x,t,Dose)c(1−ck)︸Cell loss  due  to  radiotherapy,R(x,t,Dose)={0,for  t∉therapy,(1−e−(αD+βD2)),for  t∈therapy,

where *D* and *k* denote the dose and tumour carrying capacity, respectively. *R*(*x*, *t*, Dose) is the probability of death of cancer cells (one minus cell survival fraction given by the linear-quadratic model of cell survival (*S* = *e* ^−(*αD*+*βD*^2^)^)) due to radiotherapy.

In previous models, passive translocation of cells due to ECM-cell interactions and active cell migration were overlooked. Retaining reaction-diffusion formula as the framework, Tracqui [16] introduced the effects of passive translocation of cells due to ECM-cell interactions and active cell migration up to adhesivity gradient. The variables *u*, *ρ*, and *c* were designated for mechanical displacement of cell-ECM composite, density of ECM, and cell density, respectively. The parameter *r* denotes the proliferation rate of cancer cells. Thus the reaction-diffusion formula (cell conservation equation) takes the bllowing form:

(12) ∂c∂t=−∇·(Jc+Jd+Jh)+rc(1−c),Jd=−D∇c  (diffusion term),Jc=c∂u∂t  (convection term),Jh=hc∇ρ.

The convection term addresses ECM displacement due to cells convection with velocity ∂*u*/∂*t*. Equation (12) indicates that the two new terms inhibit tumour growth. Moreover, the conservation of ECM density reads as

(13) $$\begin{array}{c} \frac{\partial \rho }{\partial t}=\underset{\text{convection}}{\underset{︸}{-\nabla \cdot \left(\frac{\rho \partial u}{\partial t}\right)}}+\underset{\text{ECM}\;\;\text{biosynthesis}}{\underset{︸}{S\left(c,p\right)}}-\underset{\text{ECM}\;\;\text{degradation}}{\underset{︸}{G\left(c,p\right)}}, \end{array}$$

where *S*(*c*, *p*) and *G*(*c*, *p*) denote the rate of formation and loss of ECM, respectively. For the sake of simplicity, ECM turnover was neglected, that is, *S*(*c*, *p*) = *G*(*c*, *p*) = 0. Thus (12) and (13) together with the equation regarding viscoelastic response of ECM to cells' traction force formed a set of differential equations for modelling. Nonhomogeneous and nonsymmetric profile at the tumour surface was obtained by the model. To validate the model, it was suggested to compare growth pattern generated by the model with that acquired from *in vitro* experiments. To our knowledge, no article addressing such a comparison associated with this model has been found. Synthesis and degradation of ECM which was neglected in primary calculation could be further included.

More recently, the interactions of cell-cell and cell-ECM were considered in a more elaborate way in reaction-diffusion models. Gerisch and Chaplain [6] developed an analytical Partial Differential Equation (PDE) model to simulate tumour growth and invasion both one and two dimensionally. In the study of Gerisch, firstly a local continuum model was formulated based on the system of reaction-diffusion equations proposed by Anderson et al. [21]. It was assumed that the movement of the cells is due to random motility with constant diffusion coefficient *D*_1_ (assuming constant ECM density), and haptotactic response to the ECM gradient. As a matter of fact, cancer cell motility depends on both ECM gradient and density, thus this was a simplifying assumption. The series of differential equations constituting the model are as follows:

(14) ∂c∂t=∇·[D1∇c−χc∇ν]+μ1c(1−ϑ1c−ϑ2ν),∂v∂t=−γmv+μ2(1−ϑ1c−ϑ2ν),∂m∂t=∇·[D3∇m]+αc−λm,

where *c*(*x*, *t*), *v*(*x*, *t*), *m*(*x*, *t*) denote the cancer cell density, the ECM density and the concentration of Matrix Degrading Enzyme (MDE), respectively. The parameters *ϑ*_1_ and *ϑ*_2_ are fractions of unit volume occupied by cancer cells and ECM, respectively. *μ*_1_, *μ*_2_, *γ*, *D*_3_, *α* and *λ* denote proliferation rate of cancer cells, remodelling rate of ECM, degradation rate of ECM, MDE diffusion coefficient, the rate of release, and removal of MDE, respectively. Finally, *χ* is designated for haptotactic function. Equation (14) differs from that of Anderson in two aspects: Employing logistic proliferation and applying modified haptotactic function to prevent cellular overcrowding at boundaries. There is also a slight difference in definition of Initial Conditions (IC) associated with ECM. 

In the second step, Gerisch modified this model (14) to a nonlocal continuum model to include cell-cell and cell-ECM adhesion. To this end, the haptotactic term was substituted with a nonlocal flux term in (14). The nonlocal term represents the velocity of cancer cells due to cellular adhesion (cell-cell adhesion) and to the ECM (cell-ECM adhesion). The growth profile was simulated for both local and nonlocal models and surprisingly the detachment of a cluster of cells that degrades ECM on its way and migrates was obtained. 

Within the realm of continuum modelling, the approach that regards a tumour as a continuum medium whose overall dynamic and morphology is dependent on the microenvironment material concentration is reflected in some other works in literature [37–46]. In these models, the concentration of microenvironment materials such as nutrition supply, like oxygen and glucose, and growth inhibitor, which is either anticancer drugs or chemicals produced by immune system, is assumed to influence individual cells phenotype.

### 2.2. Hybrid Models

The above-addressed models, both deterministic reaction-diffusion equations whose solutions is in the form of invading travelling waves of cancer cells and mechano-cellular formalism (e.g., Tracqui, 1995 [16]) provide spatio-temporal spread of tumour at macroscopic level. However, the behaviour of tumours at cellular and sub-cellular levels, which becomes important when individual cell effects dominate in the course of tumour growth and invasion, such as the spatio-temporal evolution of tumour cell heterogeneity, cannot be predicted by these modelling approaches [47, 48]. Therefore, the continuum modelling is appropriate for studying systems at a large scale. Discrete modelling can overcome this limitation since it can track individual cells and update their states at each time step. Thus it is an appropriate tool to investigate the interaction between cells and ECM, phenotypic transitions of cells which leads to a nonlinear cancer system to another state that in return affects the overall behaviour and morphology of tumours and so forth. The important drawback of discrete modelling is its increasingly high computational demands as the number of cells being modelled increases. An alternative to these scale-specific models is a multiscale approach that refers to the models that contain more than one spatial and temporal scale to take into account cross-scale mechanisms in the course of tumour growth and evolution [49]. This approach is classified as “hybrid” modelling. A hybrid model comprises of a continuum deterministic part that controls the concentration of ECM and chemicals, and a stochastic discrete part governing cell migration and interactions.

Such a hybrid model of tumour growth and invasion was developed by Anderson [19]. The formalism of hybrid modelling enables to simulate specific cell processes (e.g. proliferation and cell-cell adhesion) and also inclusion of different tumour cell phenotypes at cellular level in a continuum chemical/ECM surrounding. The model parameters consisted of concentration distributions of tumour cell (*n*), ECM (*f*), MDE (*m*), and oxygen (*c*). The interaction of these parameters was represented in a set of differential equations, as follows:

(15) ∂c∂t=Dc∇2c⏞oxygen  diffusion+βf⏞oxygen  production−γn⏞oxygen  uptake−αc⏞oxygendecay,∂m∂t=Dm∇2m⏞MDE  diffusion+μn⏞MDE  production−λm⏞MDE  decay,∂f∂t=−δmf⏞ECM  degrdation,∂n∂t=Dn∇2n⏞random  motility−χ∇·(n∇f)⏞haptotaxix.

As indicated in the first line of (15), oxygen is diffused into the ECM, consumed by tumour and decayed naturally at rates *γ* and *α*, respectively. The hybrid model, that follows the path of each individual cell, requires discretising the system of differential equation based on finite difference method in a given time and space steps [21]. Each point on the grid is correlated to neighbouring grids via coefficients indicating the probability of transition from that grid to another. For example, tumour cell density is expressed as

(16) $$\begin{array}{c} n_{i,j}^{q+1}=n_{i,j}^{q}P_{0}+n_{i+1,j}^{q}P_{1}+n_{i-1,j}^{q}P_{2}+n_{i,j+1}^{q}P_{3}+n_{i,j-1}^{q}P_{4}, \end{array}$$

where indices *i* and *j *represent the location and *q *specifies the time. The coefficients *P*_0_, *P*_1_,…, *P*_4_ are probabilities of transition from the grid in question to the respective neighbouring grids. Unlike purely continuum modelling, the hybrid model, being intrinsically multiscale, allows for investigation of the effect of tumour cell heterogeneity on the morphology and phenotypic diversity of invading vascular tumours (e.g., capturing the emergent property of invasive cells) [50]. In the more recent studies of Anderson et al. [51, 52], the hybrid model was used to simulate the shape of a growing tumour under homogeneous and heterogeneous matrix distribution and a phenotypically heterogeneous tumour cell population. Also, the impact of nutrient availability during tumour development on tumour morphology was examined. The models predicted that harsh microenvironment conditions lead to a tumour mass with invasive morphology (fingering margins) dominated with a few aggressive phenotypes. Other studies independently conducted *in vivo* and *in vitro *experiments to examine the role of harsh environment (e.g., hypoxia) in the invasive morphology of tumours [18, 53]. The results of their investigations corresponded to those predicted by the hybrid model. However, neither of them examined phenotypic composition of the resulting tumours, thus these experiments just partially validate the hybrid model.

Malignant tumour invasion, driven by haptotaxis, both in the form of travelling waves (continuum models) [54–56] and hybrid models [57–59], has been also modelled by others. The model developed by Anderson and Chaplain [58] was mathematically analysed by Kubo [60] to investigate asymptotic profiles of solutions. The simulated tumour cell distribution illustrated that a cluster of cells detaches from the original tumour mass and migrates further away from the tumour as the time evolves. The simulated tumour cell distribution shows an explicit detachment of a cluster of cells and qualitatively corresponds to the results of Gerisch's study [6].

The most recent work in the continuum deterministic framework is the study of Swanson [61]. In this study the Proliferation-Invasion (PI) model was developed to produce a Proliferation Invasion Hypoxia Necrosis Angiogenesis (PIHNA) model incorporating the mechanisms related to angiogenesis cascade. Three different cellular types, namely, proliferative, hypoxic, and necrotic were described mathematically in a form of three partial differential equations in which conversions of each type to others due to microenvironmental changes were included. It is known that, while tumour cells grow and invade according to their respective proliferation and diffusion rates, the microenvironment becomes harsh and leads to the production of Tumour Angiogenic Factor (TAF) by proliferative and hypoxic cells in response to the metabolic demands of tumour. It is worth noting that the rate of production of TAF by hypoxic cells is significantly higher compared to that by proliferative cells. The presence of TAF in tumour microenvironment stimulates vascularisation. These two processes were also represented in two differential equations that formed a system of five equations for modelling. The *in silico *prediction of malignant progression of tumour corresponded well with imaging (MRI) and histologic data of three GBM patients who had approximately similar size of tumour but different hypoxic and necrotic ratios on their MR images. In the context of microscopic extension, this model can predict local invasion. However, it cannot visualize those microscopic clusters of cells detached from main mass of tumour, since it overlooks migration (via haptotaxis).

Table 1 Summarizes the major analytical models of tumour proliferation and diffusion reported in the literature. 

Analytical modelling based on conservation of cells has evolved from basic models such as the one proposed by Murray's group [12–14] to very sophisticated models considering many biological mechanisms involved in tumour growth and invasion (e.g., Gerisch and Chaplain [6]). Some significant achievements regarding prediction of tumour behaviour in the course of its progression can also be obtained using this class of modelling. However, in order to obtain a realistic model, other critical characteristics of tumour cell growth are yet to be taken into account. The heterogeneity of diffusion coefficients and multilayer nature of tumours (necrotic, hypoxic, and proliferative layers) brought about by nutrient gradient exemplify the overlooked parameters. Moreover, purely analytical (continuum) modelling seems to be too inflexible to represent the biological phenomena which are intrinsically probabilistic. Therefore, what is actually favoured is not one single precise solution for a given situation provided by analytical models, but rather a probability distribution which better describes the behaviour of such systems. 

## 3. Stochastic Models

Stochastic models are guided by probability distribution. The various techniques used in stochastic modelling are dominated by Monte Carlo and Markov approaches which are generally employed in the simulation of biological systems.

### 3.1. Markov Model

Markov models are stochastic models which simulate the state of systems with time-dependent random variables possessing Markov property. A stochastic process has Markov property (or memoryless property), if the probability distribution of future states depends only on the present state and not on the preceding sequence of events. This reads mathematically as

(17) P(Xn+1=x ∣ X1=x1,X2=x2,…,Xn=xn)  =P(Xn+1=x ∣ Xn=xn),

where *X*_*i*_s are random variables having Markov property. A Markov chain is the simplest Markov model which is a chainlike random process that transforms from one state (*i*) to another (*j*) by a transition matrix whose elements are described as

(18) $$\begin{array}{c} p_{ij}=P\left(X_{n+1}=j\;|\;X_{n}=i\right). \end{array}$$

**Figure 4:**
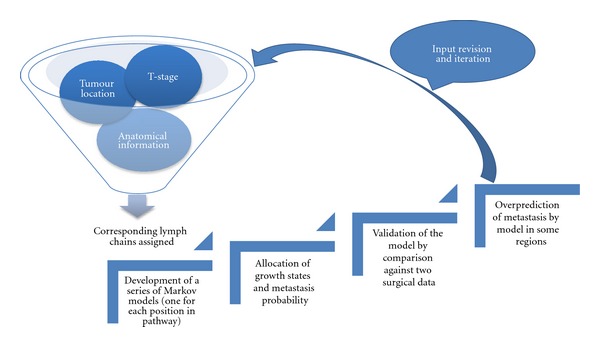
Schematic diagram of the Markov model developed by Benson et al. [3].

Benson et al. [3] produced a theoretical model to predict the microscopic spread of tumour to regional lymph nodes based on anatomical information adopted from the Foundational Model of Anatomy (FMA) in the head and neck cancer. A computational rule-based model was previously proposed in this area, based on clinical data rather than anatomical principles, by Kalet et al. [62]. FMA provides information regarding an almost complete set of drainage pathways or lymph chains which is known to be followed by subclinical spread [63]. The information acquired from FMA was supplemented by clinical data pertaining to lymph chains that span multiple regions. The inputs to the model were primary tumour location and T-stage. In FMA every primary site is associated with its respective lymphatic chains, thus lymphatic chains with subparts corresponding to the primary tumour location were derived from FMA. A sequence of Markov models were developed such that each hidden Markov model was assigned to one position in the pathway where position “0” was labelled for the original tumour. The validity of the model was examined by comparing the model results with two surgical data. Overall, the model overpredicted the metastasis in specific regions, requiring certain modifications such as revising supplementary data added to FMA. The procedure starting from model inputs to model validation followed by iteration is diagrammatically shown in Figure 4.

### 3.2. Monte Carlo Model

Monte Carlo (MC) models are widely used in the field of cancer biology and treatment since this method is particularly useful for simulating systems with considerable uncertainty in parameters.

The earliest developed MC models of tumour growth date back to early 80's, for example the work of Duchting and vogelsaenger [64] for small tumours which took into account nutritional needs of tumours. Aiming to investigate the pattern of *in vivo *cancer development, Qi [65] simulated the distribution of cancer cells in a given biochemical environment as a two dimensional cellular automaton on a square lattice. Qi et al. [66] later advanced the model to take into account proliferation of cancer cells, nutrition supply, mechanical pressure, and the cytotoxic behaviour of immune system and reproduced Gompertz model which is typically used to describe the growth of cancer tumour volume (Gompertz model of cancer tumour volume growth is *V* = *V*_0_exp⁡⁡(*A*/*B*(1 − exp⁡⁡(1 − *Bt*)), where *V* is the volume of tumour at time *t* and *V*_0_ is the initial volume. *A* and *B *are parameters). Smolle and Stettner [67] considered a two-dimensional tumour growth model and correlated macroscopic behaviour of tumour (tumour morphology) with the functionality of tumour cells at microscopic level (e.g., interaction of tumour cells with microenvironment). Later, the invasiveness of tumour in the absence of active motility was studied in a stochastic cellular automata by Smolle et al. [68]. Aimed to provide an algorithm to predict the extent and direction of spread of a brain tumour, another elegant approach was presented in a patient-specific *in vivo *brain tumour growth model which was developed by Wasserman et al. [69]. The model involved a variety of forces associated with microenvironmental (e.g., nutrient and growth inhibitor distributions) and mechanical factors (e.g., cell adhesiveness and resistance of brain parenchyma to expansion) and was implemented via the finite element method. To validate, the model was implemented on a patient MRI data to retrospectively predict the extension of tumour with respect to time. An approximate agreement between simulated tumour extension and MRI image was achieved. It is worth noting that this model explicitly addresses the problem of subclinical boundaries (CTV) in irradiation target definitions.

One of the common approaches in stochastic modelling is the Cellular Automaton (CA) method which employs a grid lattice, with each site in the grid accommodating a finite number of cells in specific states, to grow a tumour from a few cells to macroscopic stages. When the time is incremented by one, the defined biological rules determine the updated states of cells in terms of their current states and microenvironment. A 3D cellular automaton model of untreated brain tumour was developed by Kansal et al. [24, 70]. The site of tumour growth was modelled as a Delaney lattice, made of Voronoi network by connecting those sites whose polyhedra share a common face. Therefore, the density of lattice varied continuously with the radius of tumour, being greater in the centre and reduced towards the surface of the tumour. The tessellation lattice was isotropic, thus it precluded the anisotropies encountered in the models in which cubic lattice was adopted (e.g., the model presented by Duchting and Vogelsaenger [64]). However, a purely random distribution could result in some regions with either very high or very low cell density corresponding to small and large Voronoi cells, respectively. To preclude biologically unreasonable variations in size of cells, a technique called Random Sequential Addition (RSA) was used. In this technique, during the generation of random points, they are checked for not being within a given distance from neighbouring points. The tumour was proposed to be as a self-organising and ideally spherical biosystem with three different layers (necrotic, nonproliferative, and proliferative) whose thicknesses are governed by nutrition supply gradient diffusing into inner layers. This hypothesis was later supported by an *in vitro *study conducted by Deisboeck et al. [71] and was used in the model developed by Yang and Torquato [72], whereby the effect of microenvironment heterogeneity on morphology of invasive tumours was investigated. Four time-dependent variables investigated in the Kansal's model consist of overall tumour radius, proliferative and nonproliferative thickness, and probability of division. Once the lattice was generated, the initial set up was designated whereupon proliferation algorithm was applied. In the algorithm, the probability of transition of cells between nonproliferative and necrotic was considered to be a function of distance from the edge of tumour (nutrient supply) such that nonproliferative cells located at more than a specific distance from the surface of tumour were turned to necrotic. In addition, the transition between proliferative to non-proliferative occurs when there is no sufficient space for the new cell to be generated by a dividing cell. These transitions were considered stochastic in the 2D cellular automata model presented by Qi [66]. In the same framework, clonal competition (emerging a more rapidly growing tumour from a more slowly growing parent) was also quantitatively analysed by introducing another set of inputs in the model after a specific time [73]. 

**Figure 5:**
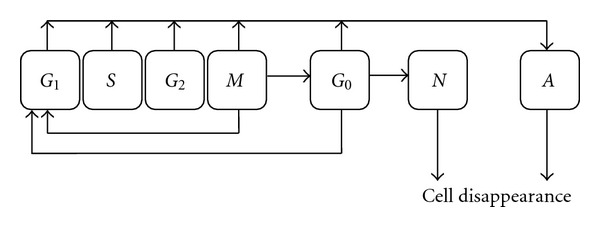
The pathway of cells through cell cycle: *G*_1_ phase (gap 1); *S* phase (DNA synthesis); *G*_2_ phase (gap 2); *M* phase (mitosis); *G*_0_ phase (if nutrition and oxygen is not sufficient, the cell enters this phase for a limited time); *N* phase (the cell enters necrotic phase, if it does not receive nutrition until the resting time is expired, otherwise it enters *G*_1_); *A* phase (apoptotic).

Aimed to simulate untreated tumour growth and also the response of tumour to different schemes of radiotherapy, a four dimensional, patient-specific, *in vivo* stochastic model was developed by Stamatakos et al. [25, 74, 75]. The model is outlined as a 3D discretising cubic mesh structure in which each mesh accommodates a specific Number of Biological Cells (NBCs) which is called a Geometric Cell (GC). In addition, different phases of tumour cell cycles have been taken into account according to the cytokinetic model proposed by Duchting et al. [76], as illustrated in Figure 5. Three metabolic subregions were considered: proliferating cell regions, resting *G*_0_ cell regions and dead cell regions. The metabolic state of each GC was determined depending on the distribution of its contained cells in different phases. The initial NBC distribution is derived from imaging and histopathological data of each individual patient, whereby the tumour region is apportioned to three metabolic layers: proliferating, resting, and necrotic. Time is discretized and at the end of each time step the GC mesh is updated such that transitions between different metabolic states are estimated and applied (e.g., M cells in a GC for which the mitosis time is over are transited to *G*_0_ or *G*_1_ with the probability depending on the subregion they belong). The time was incremented at the end of each scan and the process iterated. In order to investigate the radiotherapy effect on tumour shrinkage, the Linear Quadratic (LQ) model of surviving fraction (*S* = *e* ^−*αD*−*βD*^2^^) is employed. Three sets of radio sensitivity parameters (*α* and *β*) were assumed corresponding to proliferative, necrotic, and resting states and the tumour regression was simulated for three specific cases: standard fractionation/radiosensitive tumour, standard fractionation/moderately radiosensitive, and hyper fractionation scheme/radiosensitive tumour [77]. The simulations of tumour shrinkage under various therapeutic regimens qualitatively reproduced the clinical observations. 

The model was gradually improved to take into account possible parameters involved in tumour growth and response to radiotherapy to achieve a more biologically realistic description of cancer biology and treatment. Antipas et al. [26] studied the effect of hypoxia in radio sensitivity of tumours by introducing Oxygen Enhancement Ratio (OER) parameter and investigated the influence of OER as well as parameters corresponding to cell cycle duration on tumour growth and shrinkage under standard and accelerated fractionation regimens. The model was applied to two GBM cases, a qualitative agreement between simulation results and clinical experience was achieved. In addition, the effect of oxygen on tumour behaviour appeared to conceptually correspond to that derived by Anderson et al. [19, 50, 51]. More recently, Stamatakos et al. [27] introduced the role of neoangiogenesis distribution in a 4D model of *in vivo *tumour growth and response to radiation. In the same framework, Dionysiou et al. [78, 79] conducted parametric studies to investigate the effect of varying parameters on the radiotherapy treatment outcome with emphasis on genetic profile of tumour. Though the model includes some simplifying assumptions or may lack some parameters (since biological mechanisms in cancer are not fully understood), the discrete and modulated nature of the model allows for inclusion of further improvements. While this approach, initiated by Stamatakos et al. [25, 74] and refined later by his team [26, 27, 75, 77–79], was aimed to simulate tumour growth and response to radiotherapy, it has the potential to be improved to take into account infiltration of a malignant tumour (e.g., by introducing haptotaxis and cell-cell adhesion). This is enabled due to the discrete and modular character of the model which allows incorporation of further mechanisms without extensive modifications.

Individual-Based Modelling (IBM), which has gained popularity for modelling of biological processes, is another class of stochastic modelling [80]. In IBM approach, the biosystem population is regarded as being composed of individual cells whose sets of traits which determines their interaction with microenvironment vary. The IBM allows for explicit inclusion of variations in specifications of individual cells (heterogeneity). Aiming to investigate cancer invasion and the effect of microenvironment on growing tumour morphology and phenotype a novel IBM model was developed and further extended by Gerlee et al. [9, 30, 81, 82]. The model was constructed on a two-dimensional grid representing ECM, with each point possessing ECM, nutrition and oxygen concentration respective to that point in the ECM. Each point on the grid could either be occupied by a cancer cell or be empty. It was assumed that the cell's behaviour or phenotype is determined based on its interaction with neighbouring cells and microenvironment. Hence, a forward neural network fed with microenvironment variables as inputs to give the response of the cell (phenotype) was established. Three layers were considered for this network: (1) input layer which receives input microenvironment parameters (e.g., number of neighbours, oxygen, glucose consumption and ECM gradient); (2) hidden layer which is connected to the input layer via connection matrix consisting of regulatory genes which control the behaviour of cells via weighting factors (w) of the connection matrix; (3) output layer which is connected to the hidden layer via connection matrix (W) and determines the phenotype (e.g., metabolism, proliferation, quiescence, haptotaxis). The nutrition concentrations were modelled by reaction diffusion equations according to which concentrations were calculated for each grid at every time step (10^−1^ cell cycle). The emergence of glycolytic phenotype associated with anaerobic metabolism pathway of cells was investigated in subsequent extension of the model [81], and more recently haptotaxis was taken into account [9]. The effect of haptotaxis was included in the model by a differential equation describing degradation of ECM at grid points. Accordingly, cells take the direction with maximum ECM gradient, and when there is no gradient, the existing cells go into proliferation mode until the gradient is sufficient to move. The switch between proliferation and haptotaxis was also depended on the number of vacant neighbours. The more number of vacancies, the more probable the cell stays in proliferation mode. Finally, it was demonstrated that with the emergence of haptotaxis, tumour growth is altered showing different morphologies (compact or branched) depending on the oxygen and ECM concentration. This outcome was supported by other analyses of the model [82, 83] and conceptually corresponded to the simulation results of the hybrid IBM model of Anderson et al. [19, 51]. 

To summarize, in clinical situations, physicians propose CTVs based on their experience of the extent of malignant tumours growth. Therefore, the ability to accurately model the tumour extension at microscopic scale is highly desirable. Within the realm of stochastic modelling, a significant number of research works has been developed to contribute to the understanding of the tumour growth and invasion via a variety of classes of Monte Carlo models. However, irrespective of the class, these studies aim to gain insight into either the biology of cancer growth in general terms or the response of tumour to radiotherapy rather than the microscopic extension of tumour which is to be incorporated in CTV. Hence, there is room for investigation in this respect, in the light of information acquired from these studies.  Table 2 summarizes a few major models of tumour growth and invasion which represent various classes of Monte Carlo models.

## 4. Conclusion

An infiltrating neoplasm undergoes several stages in the course of its growth and progression and understanding of the mechanisms governing the evolution of tumour is required to deliver an appropriate therapy which results in optimal tumour control and reduced normal tissue side effects. Mathematical modelling is recognized as a great tool to facilitate this understanding. Furthermore, mathematical models provide predictions of the probable response of tumour to therapeutic regimens in a variety of circumstances, different in terms of factors such as the tumour microenvironment, and stage. In this paper, we have reviewed the evolution of mathematical modelling of tumour growth and invasion in both analytical and stochastic approaches. Analytical models are capable to describe the behaviour of tumour at macroscopic level for specific conditions; however, they fail to provide predictions at microscopic (cellular and subcellular) level. In addition, the ongoing research to enhance the limited insight into complex and dynamic cancer systems may reveal some further parameters which have to be included in models. However, analytical models are not flexible for these modifications. On the other hand, stochastic models efficiently depict the characteristic and behaviour of tumour as this class of modelling enables introducing new parameters as well as specific anatomical boundaries. Finally, we came to believe that while none of the above-mentioned models address explicitly the microscopic extension of tumour, they have the potential to be used to deduce the extent of subclinical disease which is not detected by imaging techniques. To serve this purpose, however, models have to be further modified, applying the relevant biological parameters, to become site-specific. The tumour sites that have a relatively high histopathological data available, such as prostate and gliomas can be potentially modelled and validated faster than those having little or no clinical data related to their microscopic extension.

## Figures and Tables

**Table 1:** A summary of analytical models of tumour proliferation and diffusion.

Type	Site of modelling	Incorporated mechanisms	Model validation and results	Comments	Reference
Continuum	Glioma	Random motility with uniform diffusion; exponential proliferation	N/A	Prediction of basic behaviour of gliomas (e.g., tumour cell density is a function of *ρ*/*D*)	Cruywagen et al. 1995 [14]
Continuum	Astrocytoma	Random motility with uniform diffusion; logistic proliferation; cell loss due to chemotherapy	12 CT images of a patient/agreement between model parameters and experimental data	The model is applicable for a specific course of treatment	Tracqui et al. 1995 [12]
Mechano-chemical	Multisite	Uniform diffusion; logistic proliferation; ECM-cell adhesion; haptotaxis	N/A	While important mechanisms in tumour invasion are considered, the behaviour of tumour at cellular level cannot be predicted	Tracqui 1995 [16]
Continuum	Glioma	Random motility with nonuniform diffusion; exponential proliferation	Virtual MRI image/obtaining nonisotropic invasion pattern	Rough prediction of the extent and concentration of local invasion. Applicable for tumours >1 (mm)^3^	Swanson et al. 2002, 2000 [2, 17]
Continuum	Glioblastoma	Nonuniform diffusion; exponential proliferation; mass effect	MR images/capable to simulate complex tumour behaviour	Migration and departure of cells not taken into account	Clatz et al. 2005 [10]
Continuum-Stochastic	Multisite	Random motility with uniform diffusion; haptotaxis; three-population tumour cells; heterogeneous ECM	Model predictions consistent with clinical findings [18]	Stochastic nature of the model allows to predict avascular invading tumour morphology by following individual cells with different phenotypes at each time and space step	Anderson 2005 [19]
Continuum	Glioma	Random motility with uniform diffusion; logistic proliferation; radially biased motility; shedding of invasive cell at tumour surface	The model reproduces *in vitro* experiments data	Assuming two-population tumour cells, proliferative (core) and invasive (periphery), and modelling invasive cells. Applicable for tumours <1 (mm)^3^	Stein et al. 2007 [20]
Continuum	Multisite	Random motility with uniform diffusion; logistic proliferation; ECM-cell adhesion; haptotaxis, Cell-cell adhesion	Comparison to simulation results of Anderson et al. [21]	Simplifying assumptions: uniform diffusion and that haptotaxis is independent of ECM density; the simulation is 2D	Gerisch and Chaplain 2008 [6]
Continuum	multisite	Random motility with uniform diffusion; logistic proliferation; two-population tumour cells; oxygen concentration	*In vivo* tumour growth observation	Assumption: cells could either proliferate or migrate where transition between these two classes is environment-dependent; haptotaxis not considered	Thalhauser et al. 2009 [22]
Continuum-Stochastic	Glioma	Random motility with nonuniform diffusion; logistic proliferation; two-population tumour cells; haptotaxis	The model predicts the tumour growth pattern of a clinical case	Stochastic step of the model allows for introduction of patient-specific parameters (e.g., tumour location)	Eikenberry et al. 2009 [8]
Continuum	Glioma	Random motility with nonuniform diffusion; logistic proliferation; radiotherapy	The biopsies of nine patients/the model reproduces RT response	In contrast with imaging-based RT response, this model incorporates patient-specific tumour growth kinetics to quantify RT outcome	Rockne et al. 2010 [23]

**Table 2:** Summaries of stochastic models of tumour growth and invasion.

Type	Site of modelling	Incorporated mechanisms	Model validation and results	Comments	Reference
Monte Carlo (cellular automaton model)	Brain	3D tessellation lattice grid, three-population tumour, nutrition gradient, clonal competition, intercellular mechanical stress	N/A	Since active motility is not taken into account, the tumour invasion cannot be investigated	Kansal et al. 2000 [24]
Monte Carlo	multisite	Different phases of cell cycle, three-population tumour cells, shrinkage of tumour due to radiotherapy, cubic grid	Application of the model to small cell lung cancer/qualitative correspondence to *in vitro *experiments	The microscopic extension cannot be predicted since each grid element is almost 1 mm^3^ accommodating 10^6^ cells	Stamatakos 2001 [25]
Monte Carlo	Multisite	Different phases of cell cycle, three-population tumour cells, shrinkage of tumour due to radiotherapy, cubic grid, hypoxia	Application of the model to two GBM cases/qualitative correspondence to clinical observations	The possibility to optimize radiotherapy fractionation regimens, unable to depict microscopic spread	Antipas et al. 2004 [26]
Monte Carlo	Multisite	Different phases of cell cycle, three-population tumour cells, shrinkage of tumour due to radiotherapy, cubic grid, hypoxia, neo-angiogenesis	Parametric validation against two different categories of GBM/qualitative correspondence to experiments	Generally, the discrete nature of these models allows for inclusion of other parameters	Stamatakos et al. 2006 [27]
Markov model	Head and Neck	Lymphatic drainage pathway, T-stage, tumour location	Comparison to two surgical data/over prediction of metastasis	Quantitative prediction of microscopic spread was found to be feasible	Benson et al. 2006 [3]
Monte Carlo (individual-based model)	Multisite	Three-population tumour, 2D grid, nutrition and oxygen concentration, different phases of cell cycle	Comparison to the study of Anderson [19] and also experimental results [28, 29]/good agreement	Haptotaxis is not taken into account thus tumour invasion is not depicted	Gerlee and Anderson 2007 [30]
Monte Carlo (individual-based model)	Multisite	Three-population tumour, 2D grid, nutrition and oxygen concentration, different phases of cell cycle, haptotaxis	Comparison to the study of Anderson [19] and also experiment results/good agreement	The influence of evolution of tumour cell phenotype in response to microenvironment on tumour development and progression is an important conclusion to be used in the study of microscopic extension	Gerlee and Anderson 2009 [9]
